# Lifespan-Extending Effects of Royal Jelly and Its Related Substances on the Nematode *Caenorhabditis elegans*

**DOI:** 10.1371/journal.pone.0023527

**Published:** 2011-08-09

**Authors:** Yoko Honda, Yasunori Fujita, Hiroe Maruyama, Yoko Araki, Kenji Ichihara, Akira Sato, Toshio Kojima, Masashi Tanaka, Yoshinori Nozawa, Masafumi Ito, Shuji Honda

**Affiliations:** 1 Department of Genomics for Longevity and Health, Tokyo Metropolitan Institute of Gerontology, Sakaecho, Itabashiku, Tokyo, Japan; 2 Department of Longevity and Aging Research, Gifu International Institute of Biotechnology, Naka-fudogaoka, Kakamigahara, Gifu, Japan; 3 API Company Limited, Nagaragawa Research Center, Nagarayamasaki, Gifu, Japan; 4 Computational Systems Biology Research Group, Advanced Science Institute, RIKEN, Suehiro-cho, Tsurumi-ku, Yokohama, Kanagawa, Japan; 5 Hamamatsu University School of Medicine, Handayama, Higashi-ku, Hamamatsu, Shizuoka, Japan; 6 Department of Food and Health, Tokai Gakuin University, Naka-kirinocho, Kakamigahara, Gifu, Japan; University of Washington, United States of America

## Abstract

**Background:**

One of the most important challenges in the study of aging is to discover compounds with longevity-promoting activities and to unravel their underlying mechanisms. Royal jelly (RJ) has been reported to possess diverse beneficial properties. Furthermore, protease-treated RJ (pRJ) has additional pharmacological activities. Exactly how RJ and pRJ exert these effects and which of their components are responsible for these effects are largely unknown. The evolutionarily conserved mechanisms that control longevity have been indicated. The purpose of the present study was to determine whether RJ and its related substances exert a lifespan-extending function in the nematode *Caenorhabditis elegans* and to gain insights into the active agents in RJ and their mechanism of action.

**Principal Findings:**

We found that both RJ and pRJ extended the lifespan of *C. elegans*. The lifespan-extending activity of pRJ was enhanced by Octadecyl-silica column chromatography (pRJ-Fraction 5). pRJ-Fr.5 increased the animals' lifespan in part by acting through the FOXO transcription factor DAF-16, the activation of which is known to promote longevity in *C. elegans* by reducing insulin/IGF-1 signaling (IIS). pRJ-Fr.5 reduced the expression of *ins-9,* one of the insulin-like peptide genes. Moreover, pRJ-Fr.5 and reduced IIS shared some common features in terms of their effects on gene expression, such as the up-regulation of *dod-3* and the down-regulation of *dod-19, dao-4* and *fkb-4.* 10-Hydroxy-2-decenoic acid (10-HDA), which was present at high concentrations in pRJ-Fr.5, increased lifespan independently of DAF-16 activity.

**Conclusions/Significance:**

These results demonstrate that RJ and its related substances extend lifespan in *C. elegans*, suggesting that RJ may contain longevity-promoting factors. Further analysis and characterization of the lifespan-extending agents in RJ and pRJ may broaden our understanding of the gene network involved in longevity regulation in diverse species and may lead to the development of nutraceutical interventions in the aging process.

## Introduction

Lifespan in metazoans is influenced not only by genetic factors [1], [2] but also by environmental factors, including temperature [3], [4], oxygen [5]–[7], food intake [8] and nutrition [9]–[16]. In the honeybee *Apis mellifera* L., queens live and reproduce for 1–4 years, yet hive workers, which are derived from the same diploid genome, live for only 3–6 weeks during the spring and summer in temperate climates [17]–[19]. Queens are fed throughout their lives with royal jelly (RJ), which is produced by the hypopharyngeal, postcerebral and mandibular glands of the worker bees. In contrast, workers are fed this RJ for only a short period of time during their larval stages. This scenario raises the possibility that RJ contains longevity-promoting agents for queens [17], [19]. An analysis of its chemical composition showed that RJ comprises proteins, sugars, lipids, vitamins and free amino acids [20] together with a variety of bioactive substances, including AMP N1-oxide [21], peptides [22]–[24], acetylcholine [25]–[27] and fatty acids, such as 10-hydroxy-2-decenoic acid (10-HDA) [28]. The mechanism by which RJ exerts its longevity effects on queen bees and the identities of the components that play critical roles in this process are largely unknown.

Lifespan-control mechanisms involving biological responses to hormonal or nutritional signals are remarkably conserved, even in diverse species, including nematodes, insects and mammals [2]. The effects of RJ on the extension of lifespan are likewise conserved between *Drosophila* [9] and mice [29], indicating that RJ plays the same role in disparate phyla.

In mammals, RJ has also been reported to possess a variety of pharmacological activities such as antibacterial [30], antitumor [31], anti-allergic [32], antifatigue [33], anti-inflammatory [34], [35] and immunomodulatory [36], [37] effects. RJ also induces neurite outgrowth [38], prevents dermatitis [39], hypercholesterolemia [40] and osteoporosis [41] and stimulates bone formation [42]. Protease-treated RJ (pRJ) has additional beneficial properties, including antioxidant activity [43], inhibitory effects on lipid peroxidation [44] and antihypertensive effects [45]–[47].

The nematode *Caenorhabditis elegans* has been widely used in studies on aging and longevity. It is an ideal model organism for such studies because of its relatively short lifespan (3–4 weeks) and well-established genetic pathways [1]. Using *C. elegans*, researchers have identified several compounds that are capable of extending lifespan and are derived from natural products including blueberries [48], herbs [49] and green tea [50], [51]. In the present study, we examined the effects of RJ and pRJ on the lifespan of *C. elegans* to identify the lifespan-extending agents in these substances and to understand the mechanism of their action.

## Results

### Effects of RJ on the lifespan of *C. elegans*

**Figure 1:**
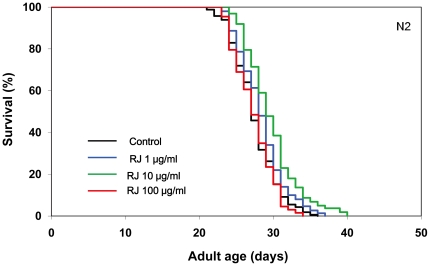
The effects of RJ on the lifespan of *C. elegans*. The survival curves of N2 hermaphrodites treated with RJ (0 (control), 1, 10 or 100 µg/ml) are shown. These substances were administered at 20°C, from the young adult stage until death. Day 0 corresponds to the L4 molt. The percentage of live worms is plotted against adult age. Detailed parameters are presented in Table S1.

**Figure 2:**
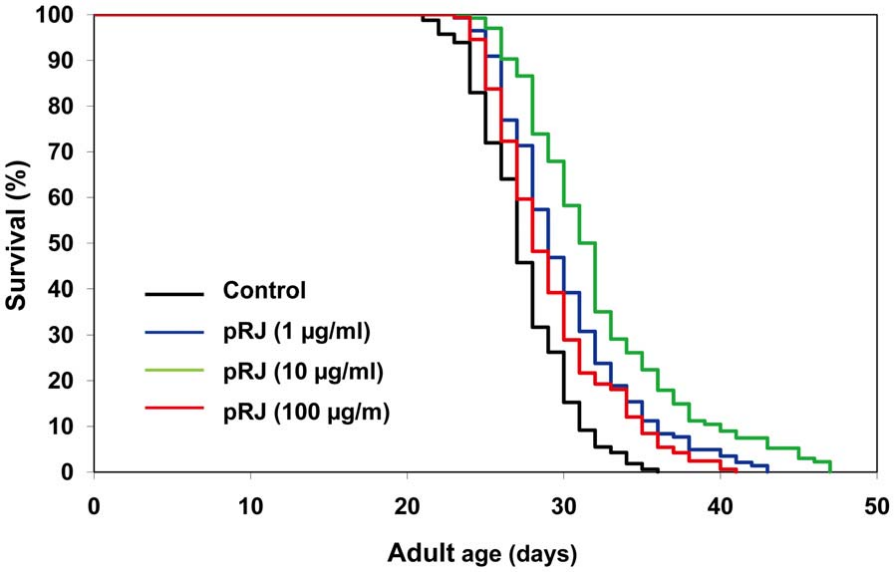
The effects of pRJ on the lifespan of *C. elegans*. The survival curves of N2 hermaphrodites treated with pRJ (0 (control), 1, 10 or 100 µg/ml) are shown. The experiment was performed as described in Figure 1 Legend. Detailed parameters are presented in Table S1.

We first investigated the effects of RJ on the lifespan of the wild-type N2 strain of *C. elegans*. RJ treatment was begun at the young adult stage with concentrations ranging from 1 to 100 µg/ml. RJ treatment at 10 µg/ml extended the mean lifespan by 7–9%, whereas either 1 or 100 µg/ml RJ had little or no effects on the lifespan (Fig. 1, Table S1), indicating that there is an optimal dose of RJ for lifespan extension. In contrast, pRJ prolonged the mean lifespan at all concentrations tested (1, 10 and 100 µg/ml). The maximal effect was observed at 10 µg/ml, at which concentration the mean lifespan was increased by 7–18% (Fig. 2, Table S1). These results suggest that both RJ and pRJ contain the lifespan-extending agents and that these agents are not proteinaceus.

**Figure 3:**
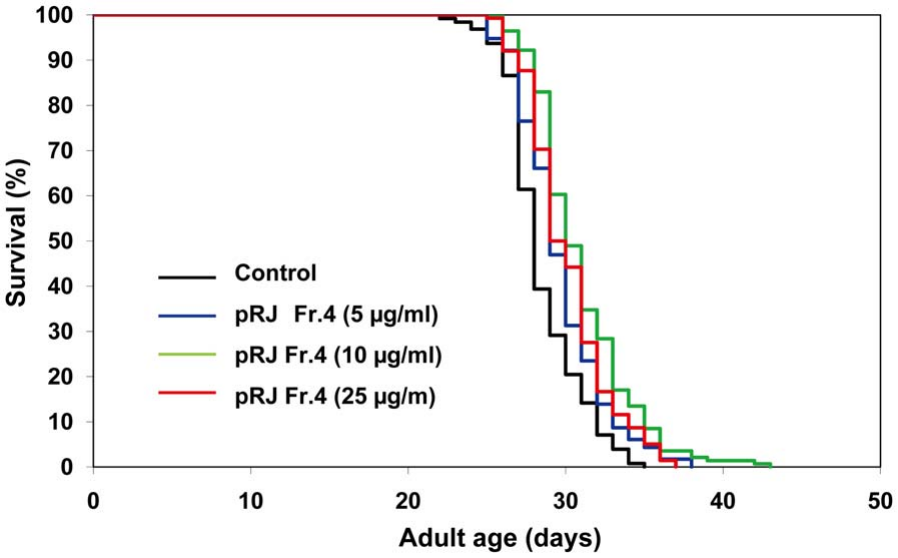
The effects of pRJ-Fr.4 on the lifespan of *C. elegans*. The survival curves of N2 hermaphrodites treated with pRJ Fr.4 (0 (control), 5, 10 or 25 µg/ml) are shown. The experiment was performed as described in Figure 1 Legend. Detailed parameters are presented in Table S1.

**Figure 4:**
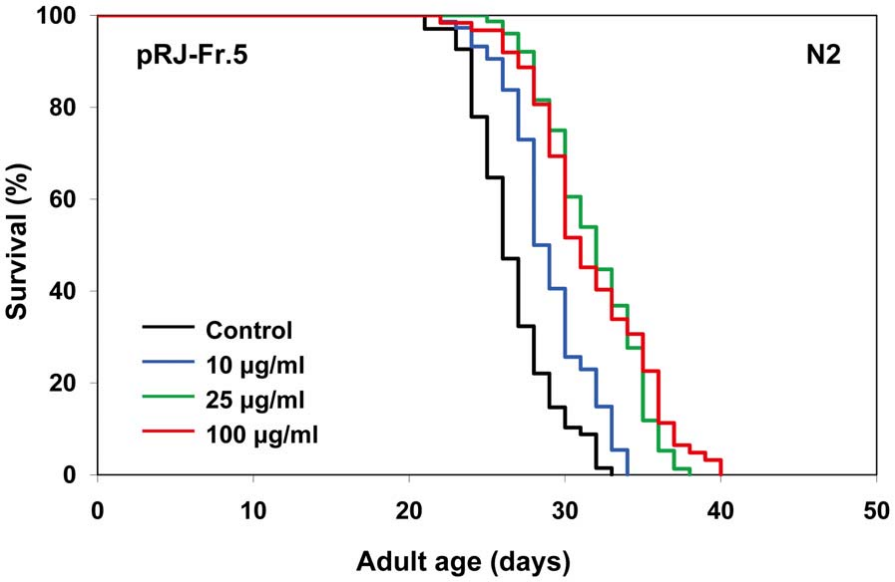
The effects of pRJ-Fr.5 on the lifespan of *C. elegans*. Survival curves of N2 hermaphrodites treated with pRJ-Fr.5 (0 (control), 10, 25 or 100 µg/ml). The experiment was performed as described in Figure 1 Legend. Detailed parameters are presented in Table S1.

To gain insights into the nature of the lifespan-extending agents, we performed fractionations of both RJ and pRJ. RJ was divided into EtOH-soluble (RJ-Fr.1) and water-soluble (RJ-Fr.2) fractions. Neither of these fractions extended the lifespan (Fig. S1, Fig. S2, Table S1). pRJ was fractionated by Octadecyl-silica (ODS) column chromatography and eluted with water (pRJ-Fr.4) and subsequently with 30% MeOH (pRJ-Fr.5). pRJ-Fr.4 at concentrations from 5 to 100 µg/ml increased the mean lifespan (Fig. 3, Table S1). The maximal effect was observed at 10 µg/ml, at which concentration the mean lifespan was increased by 9%. In contrast, pRJ-Fr.5 at concentrations of 10, 25 and 100 µg/ml increased the mean lifespan by 8–9%, 18–19% and 17–19%, respectively (Fig. 4, Table S1). These results indicated that the lifespan-extending agents in pRJ were enriched in the 30% MeOH-eluted fraction more than in the water-eluted fraction.

### Gene expression changes during pRJ-Fr.5 treatment

**Figure 5:**
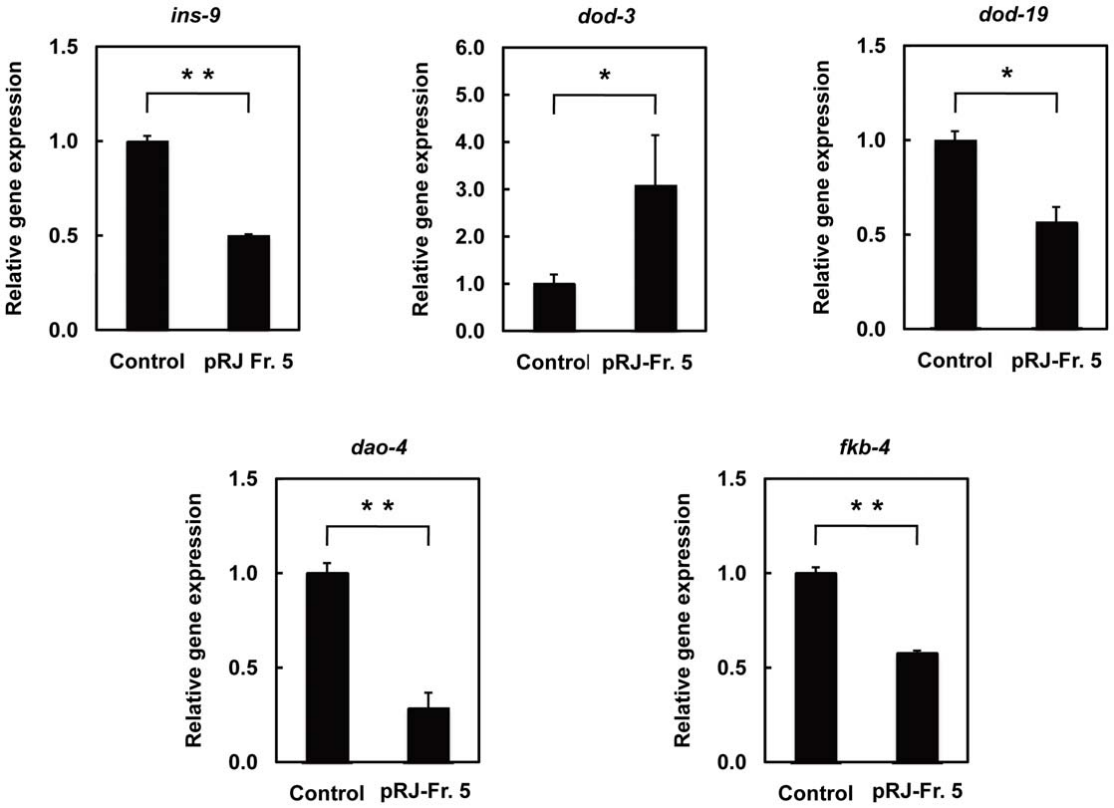
The effects of pRJ-Fr.5 treatment on gene expression in *C. elegans*. Relative expression levels of genes (*ins-9, dod-3, dod-19*, *dao-4*, and *fkb-4*) in N2 hermaphrodites treated with pRJ-Fr.5 (0 (control) or 25 µg/ml) for 24 h starting at the L4 stage. Data are expressed as the mean ± S.E. (n = 3). *: p<0.05; **: p<0.01, compared with control (Student's t test).

To understand the mechanism underlying the lifespan extension by pRJ-Fr.5, we analyzed genome-wide changes in gene expression during treatment of *C. elegans* N2 with pRJ-Fr.5. Using the Agilent *C. elegans* (V2) Gene Expression Microarray, we monitored the expression of 20,000 genes. To identify differentially regulated genes, we eliminated all probes with absent or marginal flags and then performed a *t*-test with the significance level set at *p*<0.05. Of these genes, 733 were further selected using the criterion of at least a 1.8-fold change (Table S2). Further analysis of these 733 genes revealed that pRJ-Fr.5 down-regulated *ins-9* and up-regulated *ins-20* and *ins-23*, all of which encode insulin-like peptides (Table S2). Among these insulin-like peptide genes, real-time RT-PCR confirmed down-regulation of *ins-9* gene expression after pRJ-Fr.5 treatment (Fig. 5). These results are consistent with previous findings implicating reduced insulin/IGF-1 signaling (IIS) in lifespan extension [1]. pRJ-Fr.5 also down-regulated *dod-19*, *dao-4,* and *fkb-4* and up-regulated *dod-3* (Table S2). These expression changes were all verified by real-time RT-PCR analysis (Fig. 5) and, more importantly, correlated with the changes observed when IIS is reduced in *C. elegans* [52], [53]. Certain DNA motifs were previously reported to be associated with the FOXO transcription factor DAF-16 [53], [54], the activation of which is known to promote longevity in *C. elegans* upon reduction of IIS [1]. The DAF-16-binding element (DBE: TTGTTTAC) [54] and the DAF-16-associated element (DAE: CTTATCA) [53] were overrepresented in the upstream regions of *ins-9*, *dod-3*, *dod-19*, *dao-4* and *fkb-4* (Table 1), suggesting that their gene expression is controlled by DAF-16 activity.

**Table 1:** DAF-16 promoter elements in the upstream region of genes commonly regulated by reduced IIS and pRJ-Fr.5.

Gene	Cosmid no.	DBETTGTTTAC	DAECTTATC
*dod-3*	C24B9.9	3	1
*dod-19*	ZK6.10	1	3
*fkb-4*	ZC455.10	1	1
*dao-4*	ZC373.6	1	1
*ins-9*	C06E2.8	2	2

The number of DAF-16-binding elements and DAF-16-associated elements in the 2kb upstream of each gene is shown.

### Effects of pRJ-Fr.5 on lifespan in *daf-16* deletion mutants

**Figure 6:**
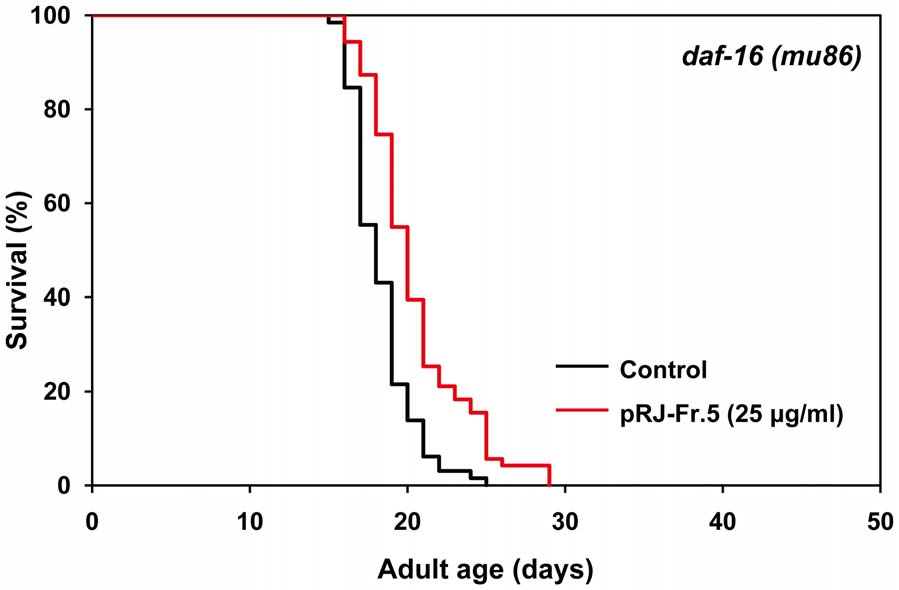
The effects of pRJ-Fr.5 on the lifespan of *daf-16(mu86)* mutants. The survival curves of *daf-16(mu86)* mutant hermaphrodites treated with pRJ-Fr.5 (0 (control) or 25 µg/ml). The experiment was performed as described in Figure 1 Legend. Detailed parameters are presented in Table S1.

To clarify whether the IIS-DAF-16 pathway is involved in pRJ-Fr.5-induced extension of lifespan, we examined the effects of pRJ-Fr.5 on the lifespan of a *daf-16* deletion mutant. The findings that pRJ-Fr.5 extended the mean lifespan of this mutant by 8–12% (Fig. 6, Table S1) and that this effect was smaller than that observed in wild-type N2 (18–19%) (Fig. 4, Table S1) indicated that pRJ-Fr.5 extends the lifespan by both IIS-DAF-16 pathway-dependent and -independent mechanisms.

### Effects of pRJ-Fr.5 on DAF-16 nuclear translocation

To ascertain whether pRJ-Fr.5 acts on the IIS-DAF-16 pathway, we examined the effects of pRJ-Fr.5 treatment on DAF-16 nuclear localization, which has been shown to be augmented when IIS is abrogated [55], [56]. We found that pRJ-Fr.5 treatment induced DAF-16 nuclear localization (Fig. S3), suggesting that pRJ-Fr.5 acts on the IIS-DAF-16 pathway.

### Analysis of pRJ-Fr.5 components

Next, we analyzed the components of pRJ-Fr.5. The amount of sugars contained in pRJ-Fr.5 was estimated to be 20%(w/w) in terms of glucose. Peptides accounted for more than 60%(w/w) of pRJ-Fr.5. The molecular weight measurement indicated that pRJ-Fr.5 contained low-molecular weight peptides, such as dipeptides and tripeptides, as well as 16.5% (w/w) 10-HDA. We also measured the 10-HDA content in RJ, pRJ and the other fractions derived from them. The 10-HDA concentrations were as follows: RJ: 1.7%, pRJ: 5.3%, RJ-Fr.1: 8.9%, RJ-Fr.2: 2.1% and pRJ-Fr.4: <0.1%. These results showed that 10-HDA was enriched especially in pRJ-Fr.5.

### Effects of 10-HDA on lifespan in N2 and *daf-16* deletion mutants

**Figure 7:**
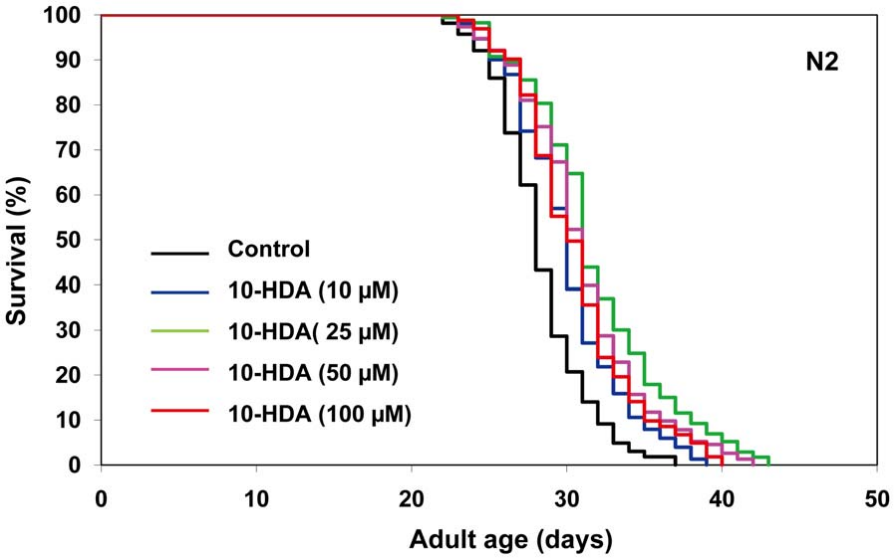
The effects of 10-HDA on the lifespan of *C. elegans*. The survival curves of N2 hermaphrodites incubated with 10-HDA (0 (control), 10, 25, 50 or 100 µM) are shown. The experiment was performed as described in Figure 1 Legend. Detailed parameters are presented in Table S3.

To elucidate whether 10-HDA is a lifespan-extending agent, we assessed its effects on lifespan. Worms treated beginning at the young adult stage with concentrations of 10-HDA ranging from 10 to 100 µM all showed extensions of the mean and maximum lifespans (Fig. 7, Table S3). The largest increase was observed at 25 µM, at which concentration the mean lifespan was increased by 12%.

**Figure 8:**
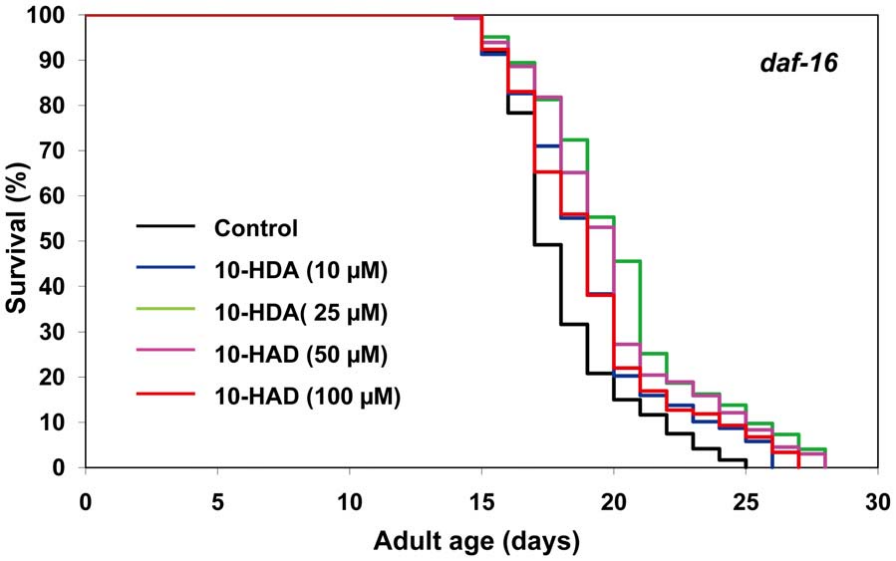
The effects of 10-HDA on the lifespan of *daf-16(mu86)* mutants. The survival curves of *daf-16(mu86)* mutant hermaphrodites incubated with 10-HDA (0 (control), 10, 25, 50 or 100 µM) are shown. The experiment was performed as described in Figure 1 Legend. Detailed parameters are presented in Table S3.

We next tested whether 10-HDA extends lifespan through the IIS-DAF-16 pathway by measuring the effects of this compound on the lifespan of the *daf-16* deletion mutants. 10-HDA at concentrations from 10 to 100 µM extended the mean lifespan of this mutant by 6–15% (Fig. 8, Table S3). The ability of 10-HDA to extend the lifespan of the *daf-16* deletion mutants and the wild-type N2 to similar extents clearly indicates that its lifespan-extending effect is mediated through a mechanism independent of the IIS-DAF-16 pathway.

### Effects of combined treatment with pRJ-Fr.5 and 10-HDA on lifespan

**Figure 9:**
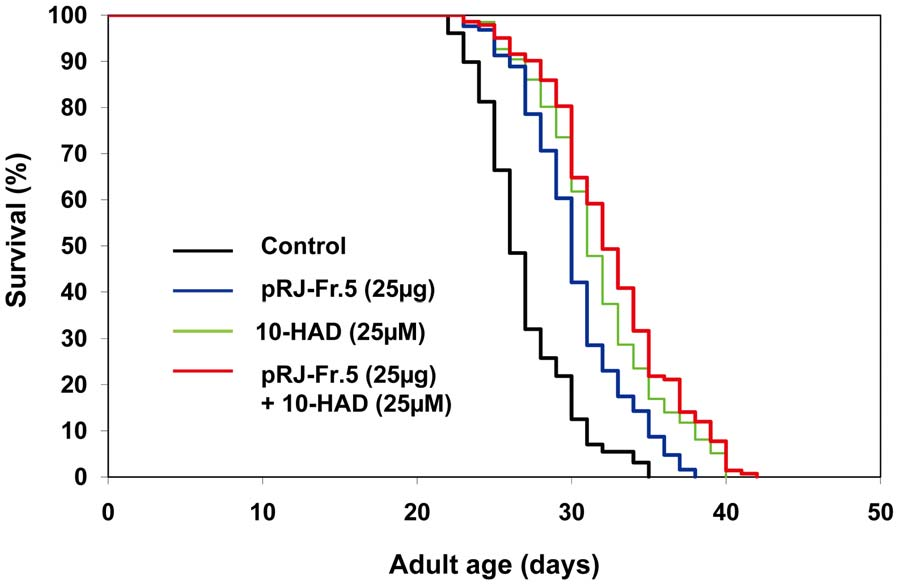
The effects of 10-HDA and/or pRJ-Fr.5 on the lifespan of *C. elegans*. The survival curves of N2 hermaphrodites incubated with 10-HDA (0 or 25 µM) and/or pRJ-Fr.5 (0 or 25 µg/ml) are shown. The experiment was performed as described in Figure 1 Legend. Detailed parameters are presented in Table S3.

To examine the contribution of 10-HDA to pRJ-Fr.5-induced lifespan extension, we tested the effect of combining pRJ-Fr.5 and 10-HDA on lifespan. The lifespan extension achieved by the combination of pRJ-Fr.5 and 10-HDA was greater than that induced by each treatment alone, but the effect was less than additive (Fig. 9, Table S3). These results suggest that pRJ-Fr.5 and 10-HDA do not extend lifespan independently of each other. Therefore, part of the lifespan extension by pRJ-Fr.5 is probably due to its 10-HDA component.

## Discussion

The present study demonstrates that RJ has the ability to prolong the lifespan of *C. elegans* (Fig. 1), as it is known to do in *Drosophila* [9] and mice [29], suggesting that RJ may contain longevity-promoting factors that can act in diverse species across phyla. This lifespan-extending activity of RJ in *C. elegans* was not diminished by protease treatment of RJ (Fig. 2), indicating that proteins in RJ are not responsible for the lifespan extension. The water-eluted fraction of pRJ (pRJ-Fr.4) had some lifespan-extending activity (Fig. 3), suggesting that water-soluble compounds, such as sugars, amino acids, vitamins or peptides including protein-proteolysis products, may have such activity. Although RJ could extend lifespan (Fig. 1, Table S1), neither the EtOH-soluble (RJ-Fr.1) nor the water-soluble (RJ-Fr.2) fraction of RJ exhibited lifespan-extending activity (Fig. S1, Fig. S2). It is unclear why this activity was not found in either RJ-Fr.1 or RJ-Fr.2. One possibility is that the concentrations of the lifespan-extending agents in RJ-Fr.1 and RJ-Fr.2 used in this study may be above or below the narrow dose range that can extend lifespan.

We found that 10-HDA extended the lifespan of *C. elegans* (Fig. 7). This is the first evidence that 10-HDA, a defined natural component of RJ, can extend organismal lifespan. 10-HDA is known to have several pharmacological activities such as antibacterial [57], antitumor [58], anti-inflammatory [59], and anti-angiogenic [60] as well as the ability to promote neurogenesis [61] and collagen production [62]. Additionally, 10-HDA is known to possess growth-inhibitory activity in honeybee queens [63]. The present observations demonstrate that 10-HDA can also perform more integrative functions, such as extending organismal lifespan.

The 30% MeOH-eluted fraction of pRJ (pRJ-Fr.5) generated by ODS column chromatography exhibited higher lifespan-extending activity than did pRJ-Fr.4 (Fig. 3, Fig. 4, Table S1). This result can be partly explained by the higher concentration of 10-HDA in pRJ-Fr.5. Furthermore, the finding that the lifespan extension induced by both pRJ-Fr.5 and 10-HDA was greater than that induced by each treatment alone but was less than additive (Fig. 9, Table S3) suggests that part of the lifespan extension by pRJ-Fr.5 was likely due to the 10-HDA contained in pRJ-Fr.5.

A variety of intricate regulatory networks have been shown to control lifespan [2]. Among them, IIS has been well established as a fundamental pathway that regulates the lifespan of *C. elegans, Drosophila* and mice [64]. It has been suggested that this pathway is a key determinant of the lifespan differences between honeybee queens and workers [65]. Reduced IIS extends the lifespan through DAF-16, a FOXO transcription factor in *C. elegans* [66]-[68]. We found that pRJ-Fr.5 induced nuclear localization of DAF-16 (Fig. S3), indicating that pRJ-Fr.5 activated DAF-16. However, our results showed that the mean-lifespan extension by pRJ-Fr.5 in N2 was greater than that in the *daf-16* deletion mutant (Fig. 3, Fig. 4, Table S1), indicating that pRJ-Fr.5 extended the lifespan by both DAF-16-dependent and DAF-16-independent mechanisms. This finding is consistent with the notion that pRJ-Fr.5 extends the lifespan in part through the IIS-DAF-16 pathway and in part through some other mechanism.

We performed DNA microarray and real-time RT-PCR analyses to identify pRJ-Fr.5-regulated genes. In these genes, *ins-9* was down-regulated by pRJ-Fr.5. Among the 40 known insulin-like peptides in *C. elegans*, INS-1 [69], INS-7 [53], INS-11 [70], INS-18 [71] and DAF-28 [72], [73] have been reported to be regulators of lifespan. Similar to *ins-1* and *daf-28, ins-9* is also expressed in chemosensory neurons such as ASI [74], which plays an important role in lifespan determination [74], [75]. Interestingly, the expression of *ins-7* has been reported to be regulated by IIS-DAF-16 [76]. We also suggested that *ins-9* expression is also controlled by IIS-DAF-16 from the finding that the DBE and DAE are overrepresented in the upstream regions of *ins-9* (Table 1).

We also found that pRJ-Fr. 5 down-regulated *dod-19*, *dao-4* and *fkb-4* and up-regulated *dod-3* (Fig. 5, Table S2), gene expression changes that are also observed when IIS is reduced [52], [53]. The *dod-19* gene encodes an unknown protein; however, intriguingly, it is one of the known determinants of lifespan [53]. It is also interesting to note that *fkb-4* encodes a homolog of the mammalian protein FKBP [52], which binds to the immunosuppressant FK506 and rapamycin. FKBP is involved in the mammalian target of rapamycin (TOR) pathway [77]–[82] and in diverse cellular functions, including protein folding and the modulation of oxidative stress [83]. FKBP also has neural roles [84], [85]. Inhibition of the TOR pathway has been found to increase lifespan in a variety of species, including yeast, nematodes, flies, and mice [86]–[91]. The deletion of both *fkb-4* and *fkb-5*, another FKBP gene, results in lethality under cold conditions [92], and it has been observed that cold conditions affect lifespan in *C. elegans* [3]. Interestingly, the TOR pathway works as an energy- and nutrient-sensing pathway to determine the queen/worker differentiation in honeybees [93]. Further research is necessary to determine whether these genes are actually involved in the lifespan extension mediated by pRJ-Fr.5.

Recent investigations have provided evidence of common longevity regulation pathways between nematodes, insects and mammals [1], [64], [87], [90], [91]. The further identification and characterization of the longevity-promoting compounds contained in RJ will broaden our understanding of the gene networks involved in longevity regulation in diverse species and may lead to the development of nutraceutical interventions in the aging process.

## Materials and Methods

### Nematode strains and culture conditions

The *C. elegans* strains were maintained at 20°C on nematode growth medium (NGM) agar with *Escherichia coli* OP50 as a food source, as previously described [94]. The N2 Bristol strain was used as the wild-type *C. elegans*. The mutant strain used in this study was CF1038: *daf-16(mu86)* I and TJ356: *zIs356* [Ex(*daf-16::gfp + rol-6)*].

### Royal jelly and protease treatment

Fresh RJ, which was produced by honeybees (*Apis mellifera* L.) foraging on *Brassica sp*. in China, was obtained from Api Co., Ltd., Gifu, Japan. RJ hydrolyzed by Protease N (pRJ) was prepared as previously described [95]. The following drugs and chemicals were purchased and used: 10-HDA (Alfresa Pharma Co., Ltd., Osaka, Japan) and Protease N “Amano” (from *Bacillus subtilis*; Amano Enzyme Inc. Aichi, Japan).

### Fractionation of RJ

Fresh RJ (1 kg) was mixed with water (1 L), hexane (2 L) and EtOH (4 L), and then shaken slowly overnight at room temperature. This mixture was filtered through No. 2 filter paper and then the extracts were concentrated under pressure until they became a dark yellow material (RJ-Fr.2). This residue was dried by heating under reduced pressure and then mixed with 5% EtOH. The supernatant from this suspension was then freeze-dried (RJ-Fr.1). The yields of RJ-Fr.1 and RJ-Fr.2 were 40.9% and 16.0%, respectively.

### Fractionation of pRJ

pRJ (20 g) was mixed with water and then chromatographed on an ODS column. The column was eluted stepwise with water and 30% (v/v) aqueous MeOH. Each fraction (1 L each) was collected and freeze-dried. These fractions were designated as pRJ-Fr.4 (15 g in the H_2_O phase) and pRJ-Fr.5 (3.8 g in the 30% MeOH phase).

### Determination of lifespan

Eggs that were isolated with hypochlorite were placed on fresh NGM agar plates containing UV-killed *E. coli* strain OP50, unless otherwise stated. UV-killing was used to avoid any effects of live *E. coli* on the compounds examined in this study and any effects of these compounds on growth of live *E.coli*. To kill the OP50, plates covered with OP50 were UV-irradiated as previously described [96]. Worms were raised until the L4 molt and were subsequently transferred onto a new plate containing 40 µM 5-fluoro-2′-deoxyuridine (FUdR, Sigma Aldrich, St. Louis, MO, USA) to prevent self-fertilization. The day of transfer at the L4 molt was counted as 0-day adult in the lifespan assay. The worms were transferred to fresh plates daily, and the number of surviving worms was monitored until death unless otherwise stated. Worms were judged to be dead when they did not respond to a mechanical stimulus. To focus on aging, worms that had become desiccated on the side of the plate after crawling off, that displayed extruded internal organs or that died because of progeny hatching inside the uterus (matricidal death) were excluded from our analysis. The results of the survival assays were analyzed using the Kaplan-Meier method, and significance was measured with the log-rank test using the statistical analysis package StatMate III (ATMS, Tokyo, Japan).

### Treatment with compounds

EtOH solutions of RJ, pRJ and RJ-Fr.1, aqueous solutions of RJ-Fr.2, pRJ-Fr.4 and pRJ-Fr.5 as well as 10-HDA in DMSO, were added to liquid NGM that had been autoclaved and cooled to 50°C. The media were immediately dispensed into Petri dishes. Experiments involving RJ, pRJ and RJ-Fr.1 were performed in parallel with those involving a control group treated with 0.1% EtOH; and experiments involving 10-HDA were conducted in parallel with those involving a control group treated with 0.03% DMSO.

### DNA microarray analysis

The *C. elegans* N2 strains were treated with pRJ-Fr.5 (0 (control) or 25 µg/ml) for 24 h beginning at the L4 stage. A total of 8,000-10,000 worms were collected for each sample. The worms were homogenized in TRIzol® Reagent (Invitrogen™, Carlsbad, CA) using a Precellys 24 (Bertin Technologies, Montigny-le-Bretonneux, France). Total RNA was extracted with a PureLink™ RNA Mini kit (Invitrogen™). The Agilent *C. elegans* (V2) Gene Expression Microarray, 4x44K (G2519F-020186) was used for global gene expression analysis. This microarray contains 43,803 *C. elegans* complementary DNA (cDNA) probes, each consisting of a single 60-oligomer oligonucleotide sequence. Target RNA labeling and hybridization were performed according to the protocol for one-color microarray-based gene expression analysis using the Quick Amp Labeling Kit (Agilent Technologies, Santa Clara, CA). In brief, 500 ng of RNA was transcribed using the oligo(dT)-based T7 promoter primer and MMLV-RT in the first- and second-strand cDNA synthesis reactions. The double-stranded cDNAs were used as templates for the preparation of fluorescent complementary RNAs (cRNAs) in the presence of T7 RNA polymerase and cyanine 3-CTP dye in an *in vitro* transcription reaction. The labeled cRNAs were purified, fragmented, and hybridized to microarrays in a rotating hybridization oven at 10 rpm for 17 h at 65°C. After hybridization, the microarrays were washed according to the manufacturer's instructions and scanned using an Agilent DNA Microarray Scanner with Scan Control software (Agilent Technologies). The resulting images were processed, and the raw data were collected using the Agilent Feature Extraction software. The gene expression data were analyzed using GeneSpring GX 11 (Agilent Technologies). The signal intensity of each probe was normalized by a percentile shift, in which each value was divided by the 75th percentile of all the values in its array. The microarray data discussed in this publication have been deposited in NCBI's Gene Expression Omnibus (GEO) and are accessible through the GEO Series accession number GSE26094 (http://www.ncbi.nlm.nih.gov/geo/query/acc.cgi?acc=GSE26094). To identify the genes with biological significance, we applied flags attributed to the signal intensity of each probe, the fold change, and the Student's *t*-test values. All the data are MIAME compliant.

### Real-time RT-PCR analysis

Total RNA was reverse-transcribed to cDNA using a High Capacity cDNA Reverse Transcription kit (Applied Biosystems), and subjected to real-time PCR using the SYBR Premix Ex Taq II (Perfect Real Time) (TaKaRa) and the Thermal Cycler Dice Real Time System (TaKaRa). The following primers were used: *ins-9* forward, 5′-GGCGAGAAGAACCTTGGAAAC-3′; *ins-9* reverse, 5′-ACAGCACAGCTTAGAGAGATCCTG-3′; *ins-20* forward, 5′-TCATCATCACAGGCACAAAGG-3′; *ins-20* reverse, 5′-GCAAAATATCATCATCCGTCAGG-3′; *ins-23* forward, 5′-CAGAGCTTCACGTTCGTAGGG-3′; *ins-23* reverse, 5′-GAACAGTACTCGGTTGGACTTGG-3′; *dod-3* forward, 5′-AAGCCATGTTCCCGAATGAG-3′; *dod-3* reverse, 5′-GCTGCGAAAAGCAAGAAAATG-3′; *dod-19* forward, 5′-ACCGTTCCCAGTTTTACAGTCC-3′; *dod-19* reverse, 5′-TATTTTGAGGCGCGGATACAC-3′; *dao-4* forward, 5′-GCACATTACAAATGCTTCAAGGAC-3′; *dao-4* reverse, 5′-TGACACCCTCATCCCCATAAC-3′; *fkb-4* forward, 5′-CTATGCGAGGAATGTGTATTGGAG-3′; *fkb-4* reverse, 5′-TGGACAGTGTAATAGAGTGGCTGAC-3′; *rla-1* forward, 5′-ACCGGCGAGAAGATCGCTAC-3′; *rla-1* reverse, 5′-CGGAAGAGACAGAAGTGATGAGG-3′. The relative expression level of each gene was calculated using the comparative Ct method. Ribosomal protein, Large subunit, Acidic (P1) family member (*rla-1*) was used as an internal control gene.

### Analysis of pRJ-Fr.5 components

Sugar content was estimated by orcinol/sulfuric acid analysis. Peptide content was estimated by the Lowry method. The molecular weights of peptides were estimated by HPLC analysis on a Superdex Peptide HR 10/30 column (Pharmacia Biotech, Uppsala, Sweden). The 10-HDA content was determined using HPLC with a Consmosil 5C18-MS-II column (Nacalai Tesque, Tokyo, Japan) at 40°C. The column was eluted with a mobile phase of 10 mM phosphate buffer (pH 2.5) and MeOH (1960:1540, v/v) at a flow rate of 1.0 ml/min.

## Supporting Information

Figure S1 The effect of RJ-Fr.1 on the lifespan of *C. elegans*. The survival curves of N2 hermaphrodites incubated with RJ-Fr.1 (0 (control), 10, 25 or 100 µg/ml) are shown. The RJ-Fr.1 was given at 20°C from 0-day adult until death. Day 0 corresponds to the L4 molt. The percentage of live worms is plotted against adult age. Detailed parameters are presented in Table S1.
(TIF)

Figure S2 The effect of RJ-Fr.2 on the lifespan of *C. elegans*. The survival curves of N2 hermaphrodites incubated with RJ-Fr.2 (0 (control), 10, 25 or 100 µg/ml) are shown. The RJ-Fr.2 was given at 20°C from 0-day adult until death. Day 0 corresponds to the L4 molt. The percentage of live worms is plotted against adult age. Detailed parameters are presented in Table S1.
(TIF)

Figure S3 The effect of pRJ-Fr.5 treatment on DAF-16 nuclear translocation. DAF-16 nuclear translocation was examined in a *daf-16::gfp* transgenic N2 line (*zIs356,* TJ356). The animals were treated with pRJ-Fr.5 (0 or 25 µg/ml) for 24 hrs. [A] Images of DAF-16::GFP animals. The arrowheads show the nuclear localization of DAF-16::GFP. [B] The translocation of DAF-16::GFP into the intestinal nuclei was not seen in untreated animals, whereas a fraction of pRJ-Fr.5-treated animals exhibited DAF-16::GFP translocation. The percentage of animals with nuclear-localized (black) and diffuse (gray) DAF-16::GFP in intestinal cells is shown. N = 58 (untreated) and 102 (pRJ-Fr.5 treated).
(TIF)

Table S1 Effects of RJ or pRJ on the lifespan.
(XLSX)

Table S2 Differentially regulated genes by pRJ-Fr.5 treatment.
(XLSX)

Table S3 Effects of 10-HDA and/or pRJ-Fr.5 on the lifespan.
(XLSX)
