# Supplementary figures and images for: Lifespan-Extending Effects of Royal Jelly and Its Related Substances on the Nematode Caenorhabditis elegans
Source: PLoS One. 2011 Aug 9;6(8):e23527. doi: 10.1371/journal.pone.0023527 (PMC3153499; doi:10.1371/journal.pone.0023527)

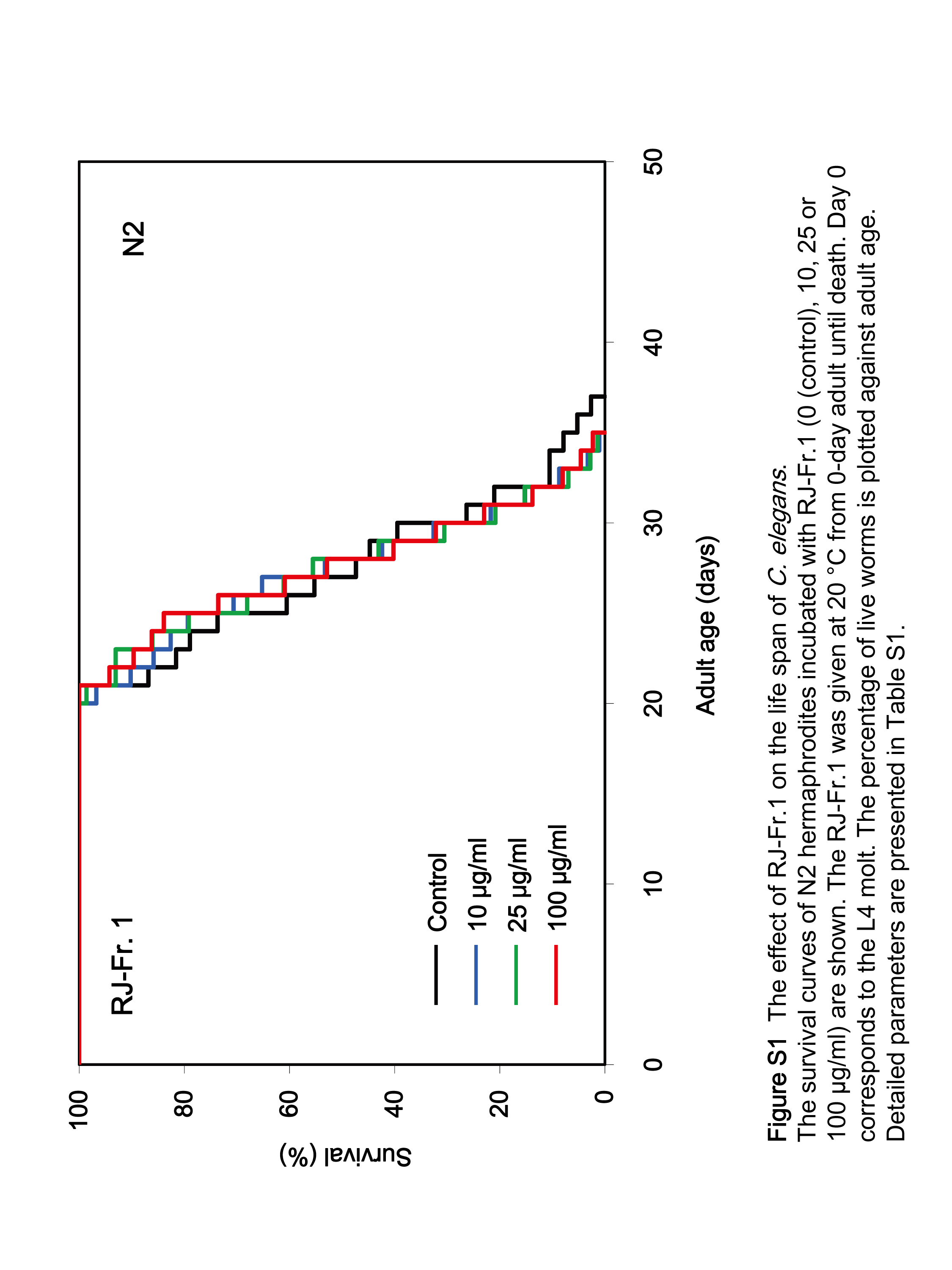

Supplement: Figure S1 — The effect of RJ-Fr.1 on the lifespan of C. elegans. The survival curves of N2 hermaphrodites incubated with RJ-Fr.1 (0 (control), 10, 25 or 100 µg/ml) are shown. The RJ-Fr.1 was given at 20°C from 0-day adult until death. Day 0 corresponds to the L4 molt. The percentage of live worms is plotted against adult age. Detailed parameters are presented in Table S1. (TIF) [file pone.0023527.s001.tif]

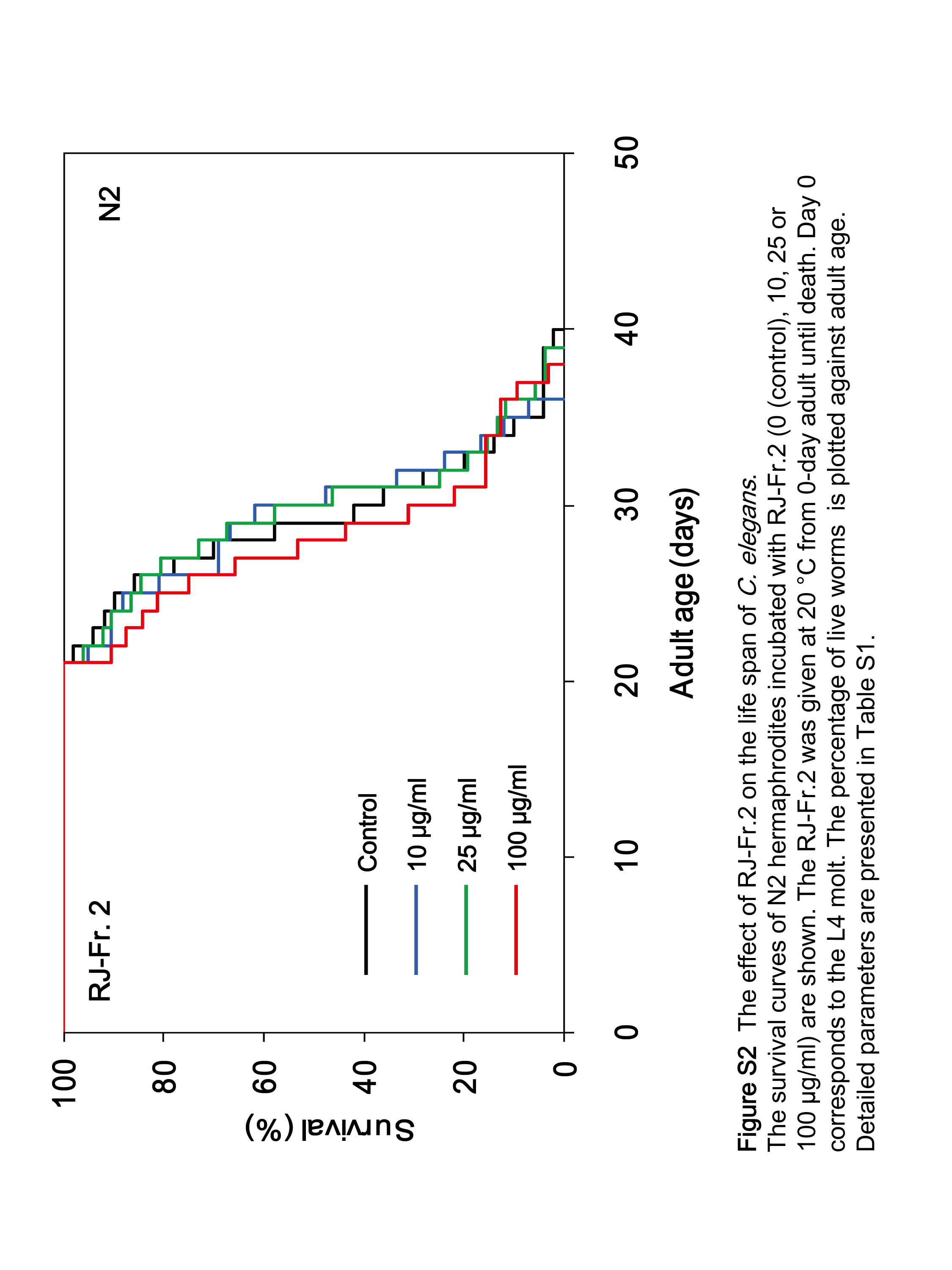

Supplement: Figure S2 — The effect of RJ-Fr.2 on the lifespan of C. elegans. The survival curves of N2 hermaphrodites incubated with RJ-Fr.2 (0 (control), 10, 25 or 100 µg/ml) are shown. The RJ-Fr.2 was given at 20°C from 0-day adult until death. Day 0 corresponds to the L4 molt. The percentage of live worms is plotted against adult age. Detailed parameters are presented in Table S1. (TIF) [file pone.0023527.s002.tif]

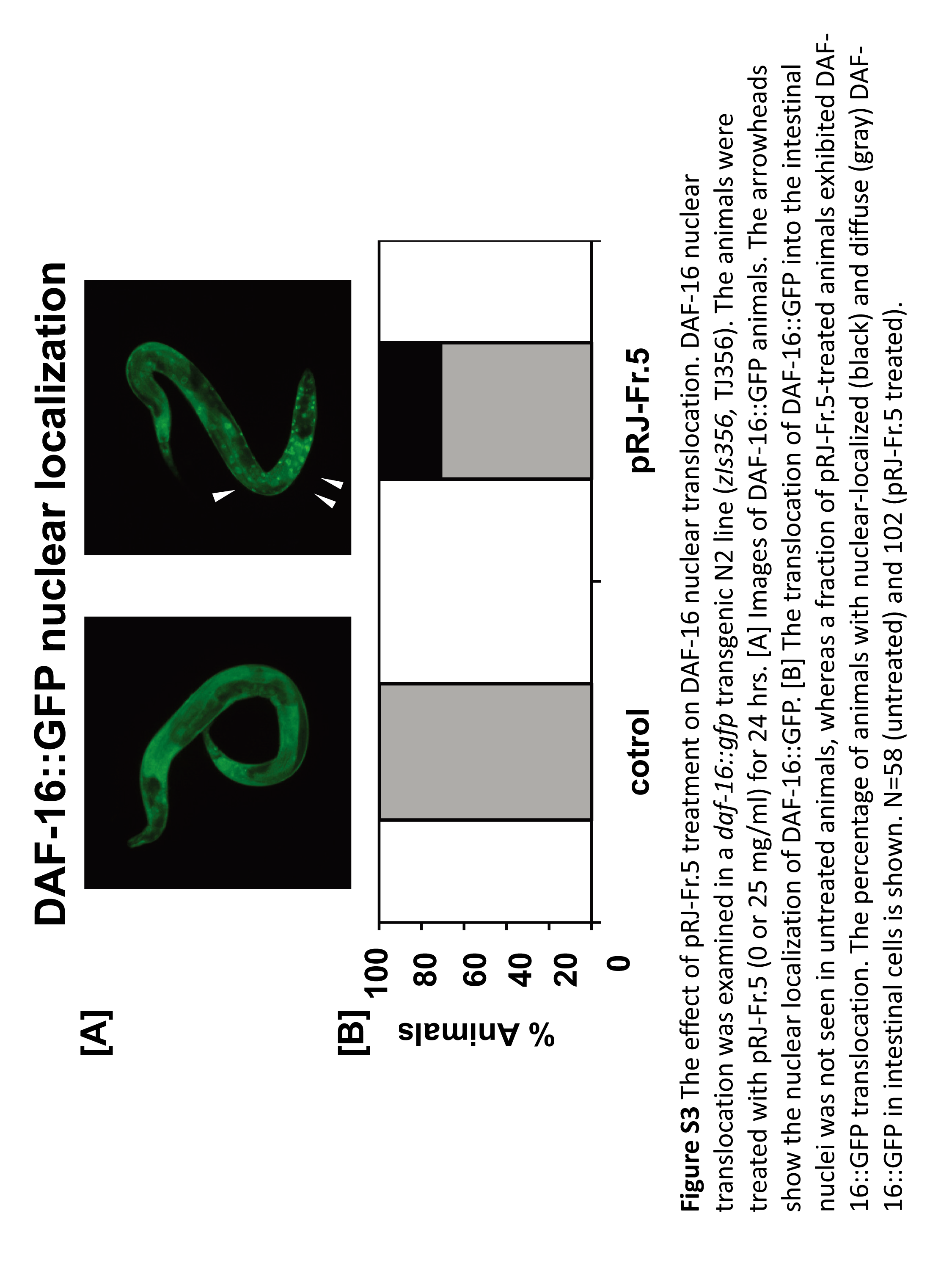

Supplement: Figure S3 — The effect of pRJ-Fr.5 treatment on DAF-16 nuclear translocation. DAF-16 nuclear translocation was examined in a daf-16::gfp transgenic N2 line (zIs356, TJ356). The animals were treated with pRJ-Fr.5 (0 or 25 µg/ml) for 24 hrs. [A] Images of DAF-16::GFP animals. The arrowheads show the nuclear localization of DAF-16::GFP. [B] The translocation of DAF-16::GFP into the intestinal nuclei was not seen in untreated animals, whereas a fraction of pRJ-Fr.5-treated animals exhibited DAF-16::GFP translocation. The percentage of animals with nuclear-localized (black) and diffuse (gray) DAF-16::GFP in intestinal cells is shown. N = 58 (untreated) and 102 (pRJ-Fr.5 treated). (TIF) [file pone.0023527.s003.tif]
